# Supplementary material for: Human RPF1 and ESF1 in Pre-rRNA Processing and the Assembly of Pre-Ribosomal Particles: A Functional Study
Source: Cells. 2024 Feb 10;13(4):326. doi: 10.3390/cells13040326 (PMC10886481; doi:10.3390/cells13040326)
Supplement: Supplementary file 1 [file cells-13-00326-s001.zip › cells-2562161-supplementary.pdf]

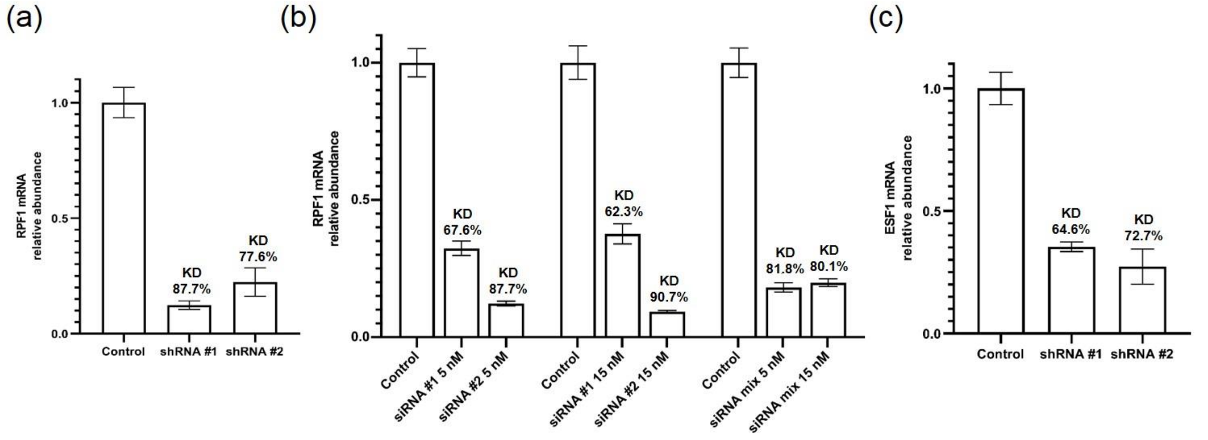

Supplementary Figure S1. RT-PCR data of RPF1 and ESF1 knockdown on their mRNA levels in HEK293 cells. **a)** RT-PCR analysis of cells stably transduced by scramble (control) or shRNAs targeting *RPF1* mRNA (shRNA #1 and shRNA #2). **b)** RT-PCR analysis of cells transfected by scramble (control) or siRNAs targeting *RPF1* mRNA (siRNA #1 and siRNA #2 separately, or mixed; 5 nM or 15 nM working concentrations of duplexes were used). **c)** RT-PCR analysis of cells stably transduced by scramble (control) or shRNAs against *ESF1* mRNA (shRNA #1 and shRNA #2).

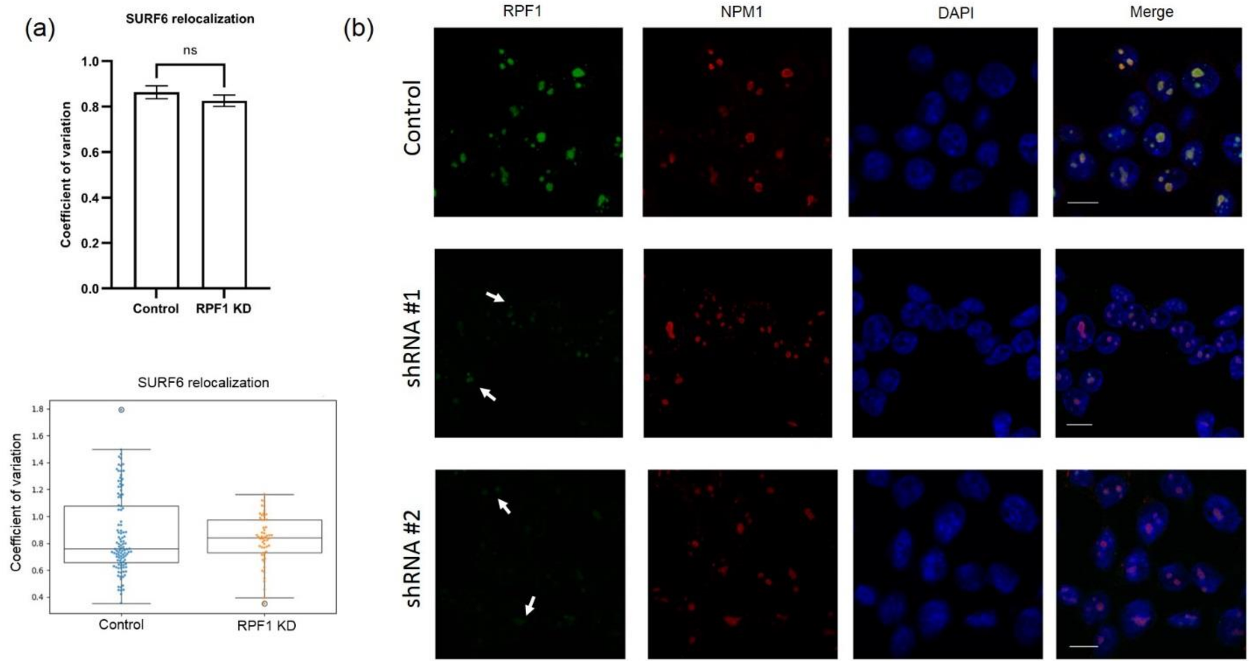

Supplementary Figure S2. Co-staining of cells with shRNA-mediated knockdown of RPF1 with anti-SURF6 and anti-RPF1. **a)** Quantifications of SURF6 relocation in RPF1 shRNA-knockdown cells relative to control cells. Coefficient of variation calculations and plotting was done as described in the Materials and Methods section. **b)** Confocal images of HEK293 cells stably transduced by scramble (control) or shRNAs targeting *RPF1* mRNA (shRNA #1 and shRNA #2). Cells were grown on cover slides, fixed, permeabilized and stained with antibodies against SURF6 and RPF1. Cells were also co-stained with DAPI to visualize nuclei. Arrows indicate the residual signals from RPF1 protein in the nucleoplasm.

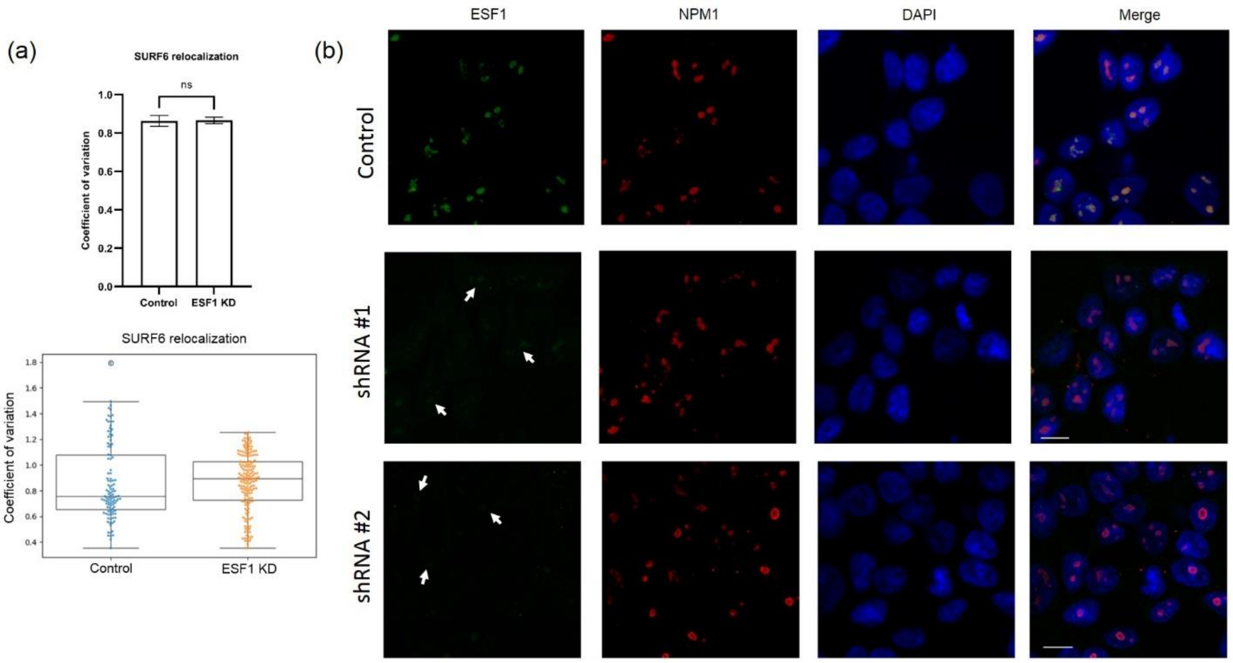

Supplementary Figure S3. Co-staining of cells with shRNA-mediated knockdown of ESF1 with anti-SURF6 and anti-ESF1. **a)** Quantifications of SURF6 relocation ESF1 shRNA-knockdown cells relative to control cells. Coefficient of variation calculations and data processing and plotting are described in the Materials and Methods section. **b)** Confocal images of HEK293 cells stably transduced by scramble (control) or shRNAs targeting *ESF1* mRNA (shRNA #1 and shRNA #2). Cells were grown on cover slides, fixed, permeabilized and stained with antibodies against SURF6 and ESF1. Cells were also co-stained with DAPI to visualize nuclei. Arrows indicate the residual signals from ESF1 protein in the nucleoplasm.

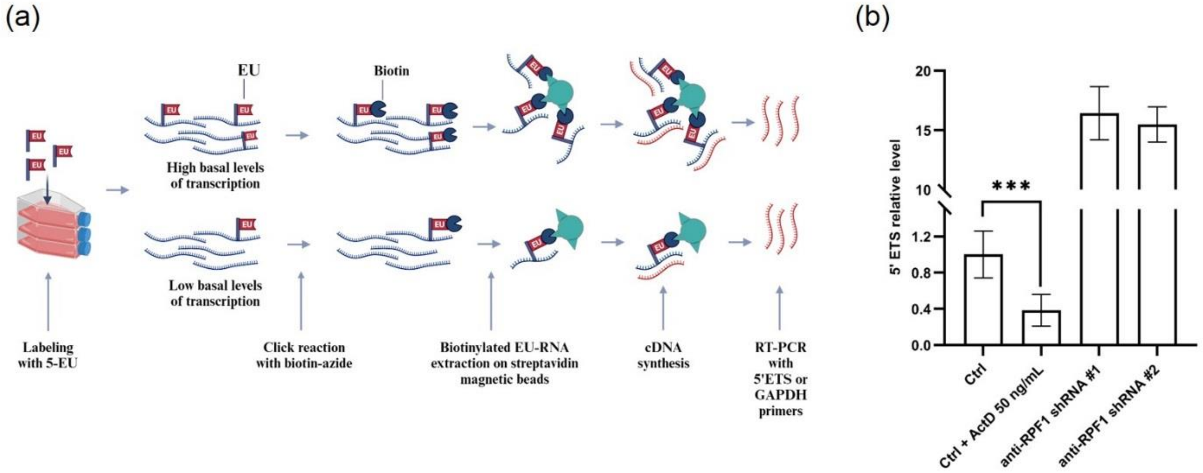

Supplementary Figure S4. Ethynyl uridine (EU) pulse labeling of cells stably transduced by scramble (control) or shRNAs targeting *RPF1* mRNA (shRNA #1 and shRNA #2). **a)** Scheme of the experiment. **b)** RT-PCR analysis of 5’ETS level in RPF1 shRNAs-knockdown cells or untreated scramble control cells, and scramble control cells treated with ActD. ActD treatment was used to inhibit PolI transcription. Mean values of three independent experiments are shown. Stars indicate statistically significant differences between control and cells treated with 50 ng/mL ActD (*** $p < 0.001$).

(a)

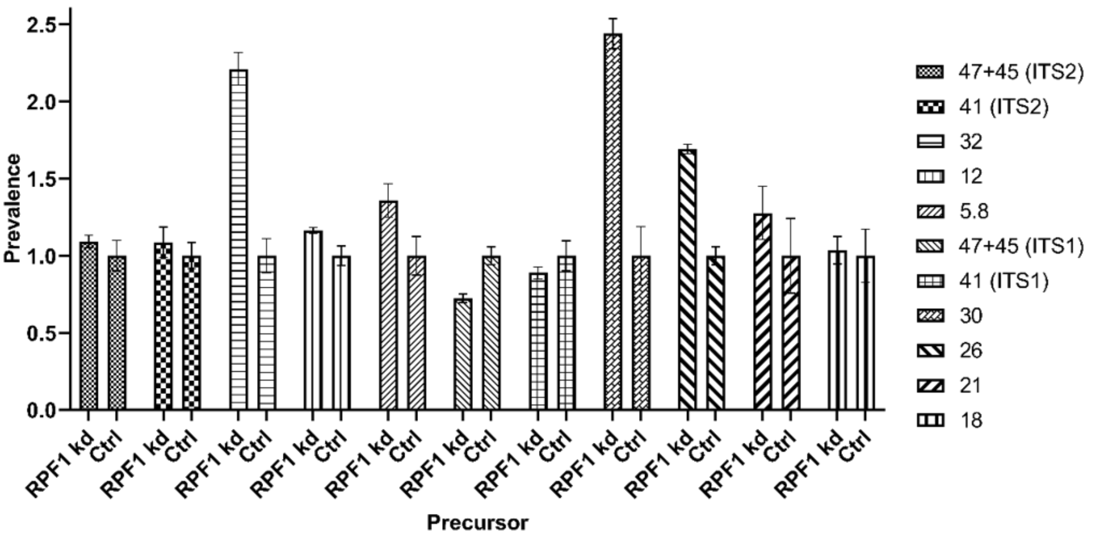

(b)

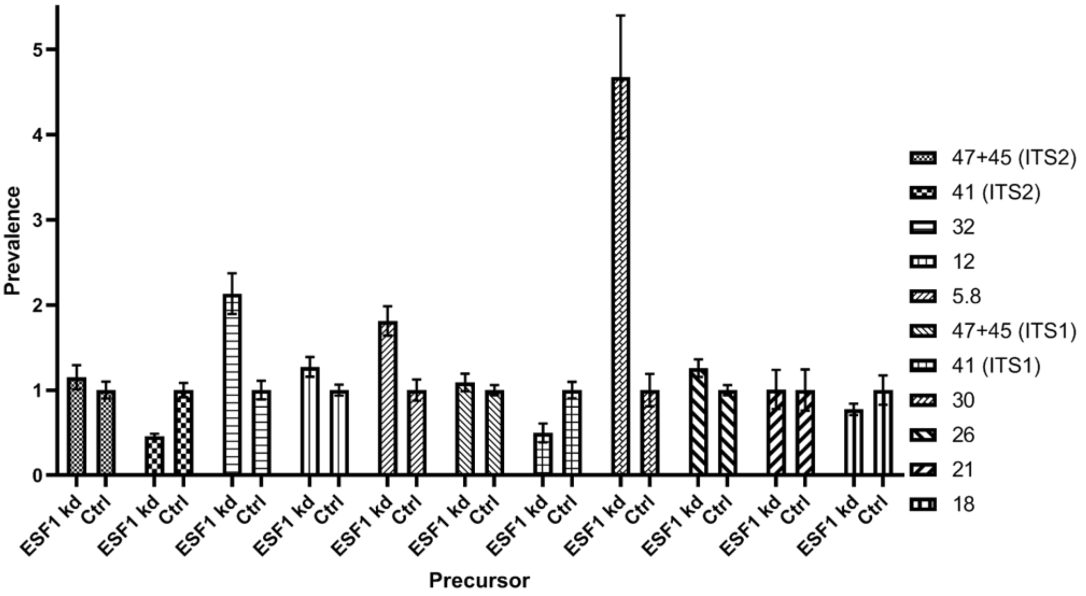

Supplementary Figure S5. Changes in the prevalence of pre-rRNA precursors in stable cell lines with RPF1 or ESF1 knockdown relative to control cells. **a)** Pre-rRNA precursors prevalence in stable cell line with RPF1 knockdown and in control cells. Mean values ± SEM of three independent experiments is shown. **b)** Pre-rRNA precursors prevalence in stable cell lines with ESF1 knockdown and in control cells. Mean values ± SEM of three independent experiments are shown.

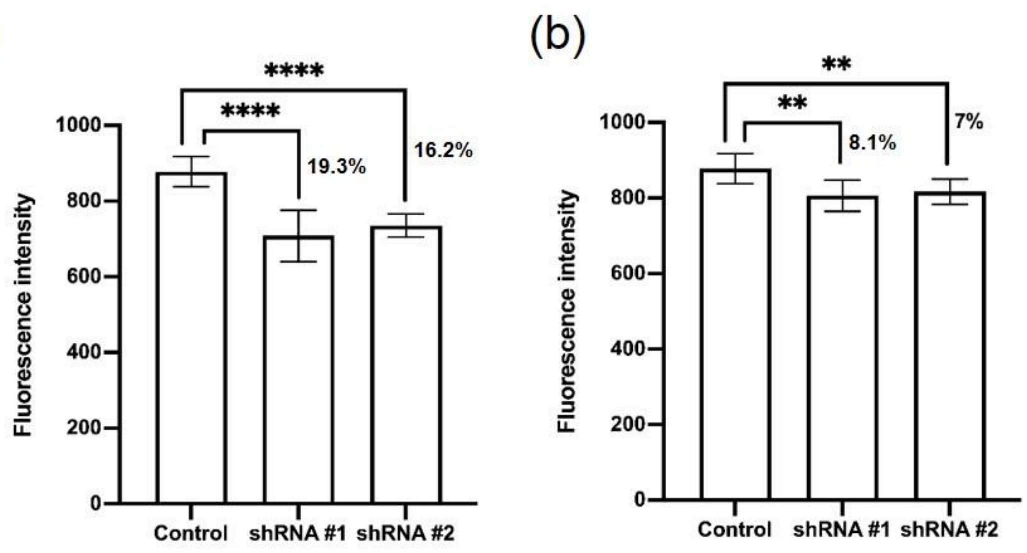

Supplementary Figure S6. Evaluation of proliferation rate of cells stably transduced by scramble (control) or shRNAs targeting *RPF1* or *ESF1* mRNAs (shRNA #1 and shRNA #2) using AlamarBlue assay. **a)** AlamarBlue assay results obtained in RPF1-knockdown stable cell line and scramble control and plotted as bar graphs. **b)** AlamarBlue assay results obtained in ESF1-knockdown stable cell line and scramble control and plotted as bar graphs. Mean OD ± SEM values at 570 nm were plotted on the vertical axis. Mean values of three independent experiments are shown. Stars indicate statistically significant differences between control and cells with knockdown of RPF1 in their viability/proliferation (** $p < 0.01$; **** $p < 0.0001$).

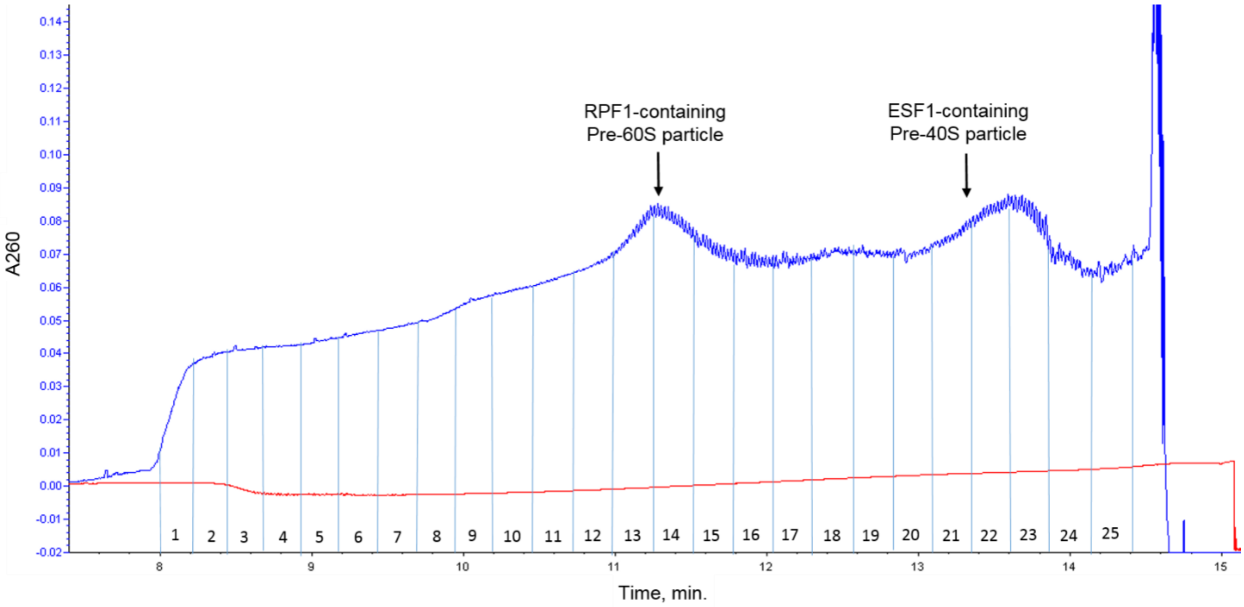

Supplementary Figure S7. Sucrose gradient profile of nucleolar fraction of wild-type HEK293 cells obtained according to the corresponding protocol in Material and Methods. Western and Northern blots from fractions are presented on the figure 7 in the main text.

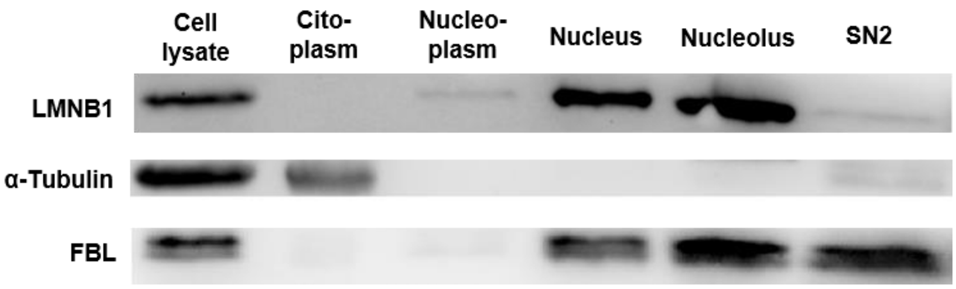

Supplementary Figure S8. Detailed analysis of nucleolus extraction and SN2 treatment procedures using Western blot. Aliquots of each compartment were prepared during extraction and processed for the total proteins preparation. Immunostaining of PAAG-separated proteins transferred to membrane and probed with anti-LMNB1 (Lamin B), anti-alfaTubulin and anti-Fibrillarin IgGs. Obtained results demonstrate that pre-ribosomal particles can be distinguish from mature ribosomes during extraction.

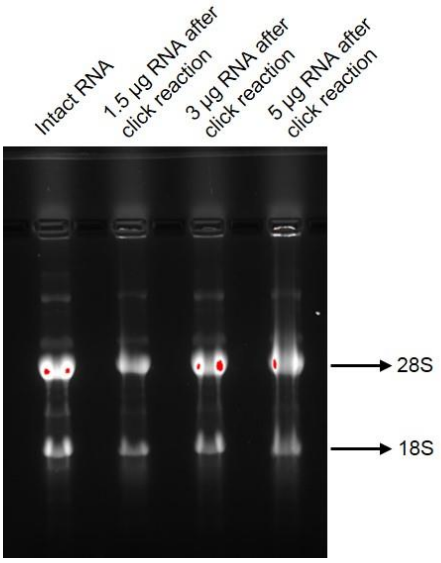

Supplementary Figure S9. Electrophoresis of RNA samples after click biotinylation in agarose gel containing 1.8 M formaldehyde (see Materials and Methods).
